# Psychedelics as a Therapeutic Opportunity or Threat: A Narrative Review

**DOI:** 10.7759/cureus.99942

**Published:** 2025-12-23

**Authors:** Paweł Liszka, Agata Ogórek, Klaudia M Olejnik-Chlewicka, Konrad Puchalski, Marta Zasiadła, Klaudia M Patrzykąt, Jakub Perediatkiewicz, Wojciech Urbański, Paweł M Łuczak, Jakub Brodowski

**Affiliations:** 1 Endocrinology, Jan Mikulicz-Radecki University Clinical Hospital, Wrocław, POL; 2 Oncology, Lower Silesian Oncology, Pulmonology and Hematology Center, Wrocław, POL; 3 Nephrology, Provincial Integrated Hospital in Kielce, Kielce, POL; 4 Nephrology, Voivodeship Specialist Hospital in Wrocław, Wrocław, POL; 5 Cardiology, Tadeusz Sokołowski University Clinical Hospital No. 1 Pomeranian Medical University (PUM) in Szczecin, Szczecin, POL; 6 Internal Medicine, 109 Military Hospital in Szczecin, Szczecin, POL; 7 Gastroenterology, New Hospital in Olkusz, Olkusz, POL; 8 Rheumatology, Jan Mikulicz-Radecki University Clinical Hospital, Wrocław, POL; 9 Paediatrics, Independent Public Healthcare Complex – Hospital in Iłża, Iłża, POL; 10 Neurology, Jan Mikulicz-Radecki University Clinical Hospital, Wrocław, POL

**Keywords:** hppd, major depressive disorder, mdma, psilocybin, psychedelic-assisted psychotherapy, psychedelics, ptsd

## Abstract

Classic psychedelics and related substances, such as 3,4-methylenedioxymethamphetamine (MDMA), have again become a focus of interest in psychiatry as potential therapeutic tools. The aim of this paper is to review current data on their mechanisms of action, clinical applications in the treatment of mood and anxiety disorders, addictions and post-traumatic stress disorder (PTSD), as well as to assess safety, drug interactions and long-term complications. Studies indicate that psychedelics act mainly through stimulation of 5-hydroxytryptamine 2A (5-HT2A) receptors, induction of neuroplastic changes and modification of functional brain networks, which may facilitate changes in entrenched cognitive and emotional patterns. Randomized clinical trials with psilocybin have shown a rapid and sustained reduction of depressive symptoms, including in populations with treatment-resistant depression and cancer patients, as well as promising results in the treatment of alcohol use disorders. MDMA, when combined with psychotherapy, has demonstrated substantial therapeutic potential in the treatment of PTSD. The literature indicates that particular caution is required when using these substances in therapy because of the risk of acute and chronic adverse reactions and potential drug interactions, including with serotonergic agents and monoamine oxidase inhibitors (MAOIs). The available findings confirm a substantial, though still only partly explored, therapeutic potential of classic psychedelics and MDMA. At the same time, they emphasize the need for strict control of “set and setting”, careful patient selection, further multicentre studies with greater statistical power and longer follow-up before these interventions can be incorporated into routine clinical practice.

## Introduction and background

Psychedelics, also referred to as classic serotonergic hallucinogens, are a group of psychoactive compounds whose mechanism of action consists of agonist or partial agonist effects at 5-hydroxytryptamine 2A (5-HT2A) receptors in the cerebral cortex, resulting in profound alterations of consciousness, perception and mood [1]. When used under controlled conditions, they are characterised by relatively low somatic toxicity and a low potential for addiction, despite their ability to induce intense subjective experiences [2]. The history of psychedelic use predates the invention of writing and is linked to their role in religious ceremonies and shamanic practices of Indigenous peoples, particularly in the Americas, where psilocybin-containing mushrooms, extracts from *Peyote* and* San Pedro* cacti and the *ayahuasca* brew were used [2,3].

Terminology and chemical classification

The term “psychedelic” derives from the Greek words *psyche* (mind, soul) and *deloun* (to reveal) and was proposed in the 1950s by Humphry Osmond to emphasise the “mind-revealing” nature of the experiences produced by these substances. The terms “hallucinogens” and “entheogens” are still used in the literature, whereas compounds with a related psychological profile, such as 3,4-methylenedioxymethamphetamine (MDMA), are described as “entactogens” or “empathogens” [2]. Contemporary approaches suggest narrowing the term psychedelics to compounds whose primary mechanism of action involves stimulation of 5-HT2A receptors, which clearly distinguishes them from dissociatives or stimulants [1].

From a pharmacological and structural perspective, classic psychedelics can be divided into three main groups: indole tryptamines (e.g. psilocybin, psilocin, N,N-dimethyltryptamine (DMT)), ergoline derivatives of lysergic acid (primarily lysergic acid diethylamide (LSD)) and phenethylamines (e.g. mescaline and related compounds) [1]. A broader class of substances with psychedelic effects also includes complex preparations such as *ayahuasca*, a decoction combining DMT with plant-derived monoamine oxidase (MAO) inhibitors, as well as substances with an entactogenic profile, chiefly MDMA, which elicit experiences that in some respects resemble classic psychedelics, although their mechanism of action is different [2,4]. This classification is reflected both in pharmacological reviews and in clinical studies focusing on individual classes of substances (Table 1) [1-5].

**Table 1:** Classification of psychedelic and related substances The table was created by the authors based on sources [1-5]. DMT: N,N-dimethyltryptamine; 5-MeO-DMT: 5-methoxy-N,N-dimethyltryptamine; 5-HT2A: 5-hydroxytryptamine 2A receptor; LSD: lysergic acid diethylamide; MDMA: 3,4-methylenedioxymethamphetamine; PTSD: post-traumatic stress disorder; MAO-A: monoamine oxidase A.

Category	Examples of substances	Predominant mechanism of action	Selected indications investigated in clinical studies
Classic indole psychedelics (tryptamines)	Psilocybin/psilocin, DMT, 5-MeO-DMT	Agonism/partial agonism at 5-HT2A and other serotonin receptors	Major depression, cancer-related depression, addictions, existential anxiety
Classic ergoline psychedelics	LSD	Potent 5-HT2A agonism with additional interactions at dopaminergic and other receptors	Studies in depression, anxiety and experimental models of altered states of consciousness
Classic phenethylamine psychedelics	Mescaline	5-HT2A agonism	Limited clinical data; mainly historical and pilot applications
Entactogens/empathogens related to psychedelics	MDMA	Marked release of serotonin, noradrenaline and dopamine with indirect activation of 5-HT receptors	MDMA-assisted psychotherapy for PTSD and other anxiety disorders
Plant-based psychedelic preparations	Ayahuasca (DMT + harmala alkaloids)	DMT: 5-HT2A and other receptor agonism; harmala alkaloids: MAO-A inhibition	Addictions, mood disorders, anxiety disorders

Regulatory history and research restrictions

Research on the use of psychedelics in Western medicine expanded rapidly in the 1950s and 1960s, when LSD and psilocybin became not only tools for studying consciousness but also promising adjuncts to psychotherapy, particularly in the treatment of addictions and depression [2]. At the same time, the rapid spread of their use outside the medical context fuelled moral panic and the geopolitical tensions of the 1960s, prompting many countries, first the United States, followed by European countries, to introduce strict legal regulations [2]. In the USA, psychedelics were classified as Schedule I substances and, in Europe, placed in the corresponding most restrictive categories of drug control, which formally implied a lack of therapeutic value, emphasised a high risk of abuse and severely limited the possibility of conducting clinical research for several decades [2].

Current perspectives in psychedelic research

Although restrictive regulations on psychedelics remain in force in many countries, a clear “renaissance” of research on these substances has been observed since the turn of the 21st century [1]. Initially, this involved small pilot studies using psilocybin to alleviate anxiety and depressive symptoms, as well as trials exploring its use in the treatment of addictions [2,6]. Over time, these preliminary experiments evolved into randomised controlled trials assessing the efficacy of psilocybin in individuals with major depression, as well as studies on psilocybin in different forms of addiction and clinical trials of MDMA-assisted therapy in patients with moderate-to-severe post-traumatic stress disorder [7-9]. In parallel, research was conducted on *ayahuasca *and other plant-based preparations, evaluated for their potential use in treating addictions and mood disorders [4].

At the same time, systematic reviews of clinical case reports and experimental studies highlight the possibility of acute psychiatric disturbances, “flashback” phenomena and persistent perceptual changes described as Hallucinogen-Persisting Perception Disorder (HPPD) [10,11]. In addition, potential interactions between psychedelics and commonly used psychotropic medications are an important consideration when selecting patients and designing therapeutic protocols [12]. Consequently, a balanced approach is required that takes into account both the promising therapeutic benefits and a careful assessment of the risk profile.

Aims and scope of this review

The aim of this narrative review is to evaluate current data on classic psychedelics and related substances (MDMA) in the treatment of selected psychiatric disorders, with particular emphasis on depression, addictions and post-traumatic stress disorder (PTSD). A further objective is to discuss neurobiological mechanisms, including neuroplastic changes and the reorganisation of functional brain networks, as well as to analyse the safety of these substances, encompassing adverse effects, long-term complications and drug-drug interactions.

## Review

Methods

The main database used to search for articles was PubMed. To obtain full-text versions of the papers, the ScienceDirect and SpringerLink platforms were also used, with Google Scholar serving as an additional search tool. The analysis covered publications from 2016 to 2025, with particular emphasis on articles published between 2021 and 2025.

Search strategies were based on combinations of keywords and the logical operators “AND/OR”. For the definitional and historical section, terms referring to the drug class itself (*psychedelic*, *psychedelic drug* in the title or abstract) were combined with terms related to the definition, etymology and origin of the term (*definition*, *etymology*, *origin*, *term*). To identify papers describing the origin of plant sources of psychedelics and their biogeography, combinations of substance names (*psilocybin*, *ayahuasca*) were used together with terms related to distribution and range (*distribution*, *geography*, *native*). For the clinical section, focusing on therapeutic applications, the search strategy combined key substance names (*psilocybin*, *LSD/lysergic acid diethylamide*, *MDMA*) with terms characterising trials with randomisation and a control group (*randomized controlled trial*, *randomised controlled trial*, *randomized*, *placebo* in the title or abstract). For the section on safety, adverse events and long-term complications, terms describing perceptual disturbances and adverse effects (*Hallucinogen Persisting Perception Disorder*, *HPPD*, *visual disturbances*, *flashbacks*, *adverse effects*) were combined with substance names (*LSD*, *psilocybin*, *MDMA*).

The review included articles published in English, specifically original studies (with a particular focus on randomised controlled trials and their long-term follow-up reports), systematic reviews and selected narrative or expert reviews, provided that they presented the current state of knowledge on mechanisms of action, clinical applications or safety of the substances in an organised way. Case reports were included indirectly, only when summarised within systematic reviews concerning adverse effects and HPPD.

Neurobiological, cognitive and psychological aspects

Molecular and Neuroplastic Mechanisms

Psychedelics display a complex pharmacological profile, but they share a high affinity for serotonin receptors, most notably 5-HT2A in the cerebral cortex. Activation of these receptors engages Gq-coupled signalling pathways, increases glutamatergic transmission and promotes changes in synaptic plasticity, among others through upregulation of neurotrophic factors such as brain-derived neurotrophic factor (BDNF) and activation of mammalian target of rapamycin (mTOR)-related signalling cascades [13]. Studies using advanced neuroimaging techniques (functional magnetic resonance imaging (fMRI), positron emission tomography (PET)) indicate that classical psychedelics such as psilocybin and LSD lead to a reorganisation of the microarchitecture and functional organisation of the prefrontal cortex, which is reflected in a greater ability of neuronal networks to switch between different activity states and to form new connections [14,15]. In a study using two-photon microscopy in animal models, a rapid and sustained increase in the number and volume of dendritic spines after administration of psilocybin was demonstrated, which is considered a potential mechanism underlying its long-lasting clinical effects despite single or short-term dosing [16].

Alterations in Brain Networks

Psychedelics induce characteristic alterations in functional connectivity at the level of brain networks. These include, among others, reduced coherence within the resting-state network dominated by the default mode network (DMN), which is involved in maintaining a sense of inner self and habitual patterns of thought, as well as a decreased hierarchical segregation between unimodal regions (processing single sensory modalities) and transmodal regions (integrating complex information and context) [15]. Studies with LSD and psilocybin also show changes in the functional connectivity of the claustrum with other brain structures, which is associated with alterations in processes underlying attention, memory and perception [14]. This “loosening” of rigid network configurations is accompanied by a more flexible mode of information processing, which may facilitate modification of entrenched patterns of thinking and emotional responding.

Perceptual, Affective and Self-Referential Changes

From a cognitive perspective, the action of psychedelics is expressed through numerous perceptual changes. Reports describe intensification of colours, the appearance of geometric patterns, moving structures and illusions of depth, as well as distortions of shapes (metamorphopsias) and object size (micropsias, macropsias). Pareidolic phenomena are common, seeing meaningful forms in chaotic visual stimuli, together with a sense that the surroundings become “animated” [2]. There may also be sharpening or distortion of sounds, changes in their timbre and in the sense of spatial location; various forms of synaesthesia are described, in which auditory stimuli evoke simultaneous visual sensations or vice versa. Such experiences are not limited to simple hallucinations but are often accompanied by a feeling that these stimuli carry profound significance, which promotes reinterpretation of one’s own experiences and relationships with the environment [17,18]. Changes in mood are also typical, depending on individual predisposition and the psychological context, ranging from inner calm, through euphoria, to anxiety and dysphoria observed in so-called challenging experiences [17].

An important feature of the psychedelic state is the modification of the sense of self. At moderate doses, one can observe a loosening of a rigid self-image, greater openness to other points of view and increased introspection. At higher doses or in specific therapeutic settings, ego dissolution may occur. Patients then describe a transient loss of clear boundaries between themselves and the external world, a feeling of “melting” into the surroundings or a sense of unity with other people, nature or abstract entities [17,19]. This phenomenon is often accompanied by elements of a mystical-type experience: a sense of going beyond time and space, deep harmony, intense emotions and the conviction of having gained insight into fundamental aspects of life. Studies with psilocybin suggest that the intensity of the mystical and ego-dissolution components is associated with long-term improvements in well-being, reductions in depressive symptoms and better social functioning, provided that adequate psychological preparation and post-session integration within psychotherapy are ensured [8,19].

Dream-Like Cognition

Psychedelic experiences also share certain features with dreaming, particularly in terms of symbolic processing of personal memories and a reduced tendency to critically evaluate ongoing mental content. Analyses highlight a similar flow of consciousness in dreams and under the influence of psychedelics, although during therapeutic sessions, at least partial capacity to monitor and evaluate one’s cognitive processes is preserved [18]. This “controlled deregulation” of consciousness may create favourable conditions for confronting difficult memories, changing the meanings attributed to personal experiences and modifying avoidance patterns, which is used, among others, in studies of therapy for depression, addictions and post-traumatic stress disorder.

Addiction Liability

From the perspective of addiction risk, psychedelics, unlike substances with a high abuse potential such as opioids or psychostimulants, do not produce strong dopaminergic reinforcement within the mesolimbic system that underlies compulsive drug-seeking. Their effects also do not favour frequent use, as the experience can be extremely intense, demanding or confrontational. After one or a few administrations, they induce marked tolerance, which rapidly attenuates the effects with short-interval repeated dosing [2]. Clinical and observational studies have not identified typical patterns of dose escalation, life organised around obtaining the substance or taking it to relieve withdrawal symptoms [20]. In therapeutic settings, psychedelics are usually given once or in a small number of sessions, and the literature suggests that they may support efforts to overcome addictions, including nicotine and alcohol dependence, by reducing craving and promoting profound motivational change [7,21,22].

Lysergic acid diethylamide (LSD)

Lysergic acid diethylamide (LSD) is a semi-synthetic ergoline derivative obtained from lysergic acid present in ergot alkaloids (*Claviceps purpurea*). It belongs to the classic serotonergic psychedelics and is characterized by very high potency at very low doses, which is mainly related to its strong affinity for 5-HT2A receptors and additional interactions with other serotonergic and dopaminergic receptors [1].

LSD was first synthesized in 1938 in the Sandoz laboratories in Basel (Switzerland) by Albert Hofmann, who was studying ergot alkaloid derivatives in the search for new vasoactive and uterotonic drugs (increasing the tone and contractility of uterine smooth muscle) [2]. Initially, the compound did not attract particular attention in the scientific community. Only in 1943, after accidental exposure to a small amount of LSD, did Hofmann experience unusual alterations of consciousness, which prompted him to carry out a deliberate self-experiment with oral administration of the substance and a detailed description of the effects [2]. This event initiated an intensive period of research on LSD as a new tool for studying consciousness and serotonergic system function.

In the 1950s and 1960s, Sandoz introduced LSD to the pharmaceutical market under the trade name Delysid (Sandoz Laboratories (Sandoz AG), Basel, Switzerland), making it available to physicians and research centres for experimental use. The compound was used both in studies of the so‑called “model psychosis” and as a potential adjunct to psychotherapy in the treatment of affective and anxiety disorders and addictions, which was reflected in a large number of clinical reports from that period [2]. Interest in the substance discovered by Hofmann was not limited to the scientific community. LSD also attracted the attention of military and intelligence services, which explored its possible use as an agent influencing behaviour or as a so‑called “truth serum” during interrogations, further fuelling controversy. The combination of non‑medical use of LSD within countercultural movements with the political concerns of the time ultimately led to its prohibition and inclusion among the most strictly controlled substances, which halted clinical research for many decades [1,2].

Psilocybin

Psilocybin is a naturally occurring indole tryptamine alkaloid, classified as a classic serotonergic psychedelic and acting mainly through its active metabolite, psilocin, which is an agonist of 5-HT2A receptors in the cerebral cortex [2]. This compound occurs naturally primarily in psilocybin‑containing mushrooms, encompassing numerous species, mainly of the genus *Psilocybe*, as well as other genera that contain related indole alkaloids [1]. In the context of clinical research, synthetic psilocybin of standardised purity is currently used [8,23].

The historical and cultural use of psilocybin is closely linked to the traditions of Indigenous peoples of Mesoamerica, particularly in the areas of present‑day Mexico and Central America. Sources describe the use of psilocybin‑containing mushrooms in religious rituals, shamanic practices and healing ceremonies, usually conducted by healers or priests [2]. In Aztec culture, they were referred to as *teonanácatl*, which in contemporary language can be rendered as “the flesh of the god”. This term emphasised their sacred status and their role as intermediaries between the human and the divine realms [2]. Reports from the colonial period indicate that mushroom ingestion was accompanied by complex ceremonies involving singing, dancing and prayer, and that the experiences under their influence were interpreted as revelations of divine will or as contact with deities or ancestral spirits [2].

Despite pressure from colonial authorities and Christian missionaries during the colonial era, some of the traditions associated with the use of psilocybin mushrooms have persisted in the practices of local shamans and continue to be observed to this day [2]. Contemporary clinical studies on psilocybin often refer to this heritage by emphasising the importance of a carefully structured therapeutic context (“set and setting”) and appropriate integration of the experience, which can be seen as a continuation of traditional roles adapted to a medical environment [8,19].

Ayahuasca

*Ayahuasca* is a complex plant-based brew in which the main compound responsible for its psychoactive properties is N,N-dimethyltryptamine (DMT). The drink combines plants containing DMT with species rich in β-carboline alkaloids (such as harmine, harmaline and tetrahydroharmine), which act as monoamine oxidase A (MAO-A) inhibitors. As a result, DMT, which when taken on its own is rapidly degraded, becomes pharmacologically active when ingested orally in the form of the brew [4].

Traditionally, the brew is prepared mainly from *Banisteriopsis caapi* vines, which are the source of β‑carboline alkaloids, and from leaves of plants containing DMT, most commonly *Psychotria viridis* [24]. The drink originates predominantly from the territories of present‑day Peru, Brazil, Colombia and Ecuador, where for centuries, it has been an important element of healing and religious practices among Indigenous communities. The name *ayahuasca *comes from Quechuan languages and is translated as “vine of the soul” or “liana of the dead”, reflecting the belief that it makes contact with the spirit world possible [3].

In cultural and shamanic contexts, *ayahuasca *continues to be used in complex ceremonies led by experienced facilitators. Their role includes preparing the brew, interpreting the content of visions and accompanying participants in the healing process. The brew is used for diagnostic and therapeutic purposes, and the experiences induced by it are interpreted as providing insight into the causes of illness, disturbances in social relationships or existential crises [4].

Current research focuses mainly on the potential of *ayahuasca *in the treatment of mood disorders and addictions. Literature reviews indicate that interventions using a standardised brew may lead to a rapid and marked decrease in the severity of depressive and anxiety symptoms, including in patients with treatment-resistant depression. These effects are partly maintained in longer-term follow-up [3]. Some studies suggest a reduction in alcohol and other psychoactive substance use, as well as a decrease in craving among individuals taking part in rituals or therapeutic programmes in which the brew is administered under controlled conditions [4]. At the same time, authors emphasise the limitations of the available data, small patient samples, lack of standardised protocols and the strong influence of the ritual context, and the need for well-designed randomised trials before *ayahuasca *can be incorporated into routine clinical practice [3].

3,4‑Methylenedioxymethamphetamine (MDMA)

3,4‑Methylenedioxymethamphetamine (MDMA) belongs to the group of synthetic phenethylamine derivatives. Natural mescaline is also classified within this group, although MDMA differs from it both in pharmacological profile and in the nature of the experienced effects. In contrast to classical serotonergic psychedelics, which act mainly as agonists at 5-HT2A receptors, MDMA primarily acts as a monoamine-releasing agent; it enhances the release of serotonin, noradrenaline and dopamine and inhibits their reuptake in synapses [9,25]. In addition, it modulates the activity of serotonin receptors (including 5-HT1 and 5-HT2). Combined with its effects on the noradrenergic and dopaminergic systems, this leads to a complex psychopharmacological profile that integrates elements of psychostimulant and psychedelic action [2]. For practical reasons, MDMA is often included in the broader category of “psychedelics” in literature on substance-assisted psychotherapy, although, in formal terms, it differs from classical representatives in both mechanism of action and the effects it produces [1,25].

MDMA is classified as an agent with empathogenic properties. It primarily induces an increased sense of connection with others, empathy and openness, a reduction in social anxiety and fear of confronting difficult emotional material, as well as an enhanced feeling of safety [25]. These effects are accompanied by symptoms typical of sympathomimetic substances, such as psychomotor agitation, increased energy and sometimes tachycardia and elevated blood pressure. Changes in visual and auditory perception are usually moderate; alterations in mood, thinking and interpersonal relating tend to dominate over overt hallucinatory phenomena [6].

MDMA was first synthesised in the first half of the 20th century as part of work on phenethylamine derivatives. However, the specific psychological properties of this compound were not widely recognised and described until the second half of the 20th century, which initiated its experimental use in the psychotherapeutic context, including for post-traumatic stress disorder (PTSD), even before the substance was placed in the group of strictly controlled psychoactive agents [2,9].

Potential therapeutic applications

Clinical trials indicate that classic psychedelics and MDMA may represent a promising therapeutic option in selected substance use disorders, severe mood disorders, and PTSD. The results of these studies are presented in Table 2 [8,9,21-23,26,27].

**Table 2:** Selected clinical trials of psychedelic and MDMA-assisted therapies The table was created by the authors based on sources [8,9,21-23,26,27]. AUD: alcohol use disorder; CI: confidence interval; p: p-value (probability value); MDD: major depressive disorder; GRID-HAMD: GRID-Hamilton Depression Rating Scale; QIDS-SR-16: Quick Inventory of Depressive Symptomatology-Self-Report (16-item version); WSAS: Work and Social Adjustment Scale; WCS: Watts Connectedness Scale; MLQ: Meaning in Life Questionnaire; HAM-A: Hamilton Anxiety Rating Scale; STAI: State-Trait Anxiety Inventory; PTSD: post-traumatic stress disorder; MDMA: 3,4-methylenedioxymethamphetamine; CAPS-5: Clinician-Administered PTSD Scale for DSM-5; BDI-II: Beck Depression Inventory-II.

Author and year	Study population	Substance/protocol	Primary assessment tool	Key outcomes
Bogenschutz et al. (2022) [22]	95 adults with severe alcohol use disorder (AUD)	Psilocybin, 2 sessions (25-40 mg/70 kg) + motivational therapy vs diphenhydramine (active placebo; 50-100 mg) + motivational therapy	Percentage of heavy drinking days over 32 weeks of follow-up, amount of alcohol consumed, abstinence indices	During the 32-week follow-up, the percentage of heavy drinking days was 9.7% in the psilocybin group vs 23.6% in the control group (difference 13.9 percentage points; 95% CI: 3.0-24.7; p = 0.01). A marked reduction in weekly alcohol intake was also observed in the psilocybin arm compared with placebo, with no serious adverse events judged to be related to the investigational drug.
Johnson et al. (2017) [21]	15 chronic smokers (≥10 cigarettes/day for ≥10 years)	Psilocybin, 2-3 sessions (20-30 mg/70 kg) + cognitive-behavioural smoking cessation therapy; no randomized control group	Abstinence confirmed by exhaled carbon monoxide (12 and >16 months), self-reported maintenance of abstinence	After 12 months, continuous abstinence was maintained by 67% of participants; at >16-month follow-up, approximately 60% remained completely abstinent. The absence of a randomised control group limits the ability to draw definitive causal conclusions.
Davis et al. (2021) [8]	27 (24 completed the protocol) adults with major depressive disorder (MDD)	Psilocybin, 2 sessions (20 mg and 30 mg) + psychological support; immediate-treatment vs delayed-treatment group	GRID-HAMD depression scale, QIDS-SR-16, response and remission rates	After 1 week, the mean GRID-HAMD score was about 8 points in the immediate-treatment group vs 23-24 points in the waitlist group. In total, 71% met the criteria for clinical response (≥50% reduction), and 54% remained in remission (GRID-HAMD ≤7) at 4 weeks.
Gukasyan et al. (2022) [27]	24 adults with MDD after psilocybin therapy (long-term follow-up of Davis et al. study population)	12-month follow-up after 2 psilocybin sessions + psychological support	GRID-HAMD, QIDS-SR-16, response and remission rates, long-term safety	At 12 months, 75% of participants still met criteria for clinical response and 58% for remission. The mean reduction in depressive symptom severity relative to baseline remained substantial, and no new safety concerns or misuse of psilocybin outside the study context were identified.
Erritzoe et al. (2024) [26]	59 adults with moderate or severe major depressive disorder (MDD)	Psilocybin, 2 sessions (25 mg) + psychological support vs escitalopram (10-20 mg/day) + psychological support	QIDS-SR-16 (primary endpoint), WSAS, WCS, MLQ and other measures of functioning and well-being	At 6-month follow-up, improvement in the primary outcome (QIDS-SR-16) was similar in both groups (between-group difference: 1.51 points; 95% CI: -1.35 to 4.38; p = 0.311). Secondary outcomes, however, favoured psilocybin therapy: greater improvements were observed in functioning (WSAS), psychological connectedness (WCS) and meaning in life (MLQ), with statistically significant between-group differences.
Griffiths et al. (2016) [23]	51 patients with life-threatening cancer and clinically significant symptoms of depression and/or anxiety	High-dose psilocybin (22-30 mg/70 kg) vs low dose (1-3 mg/70 kg) in a cross-over design + psychological support	GRID-HAMD, HAM-A, STAI and measures of quality of life and existential well-being	A high dose of psilocybin produced large, rapid reductions in GRID-HAMD and HAM-A scores. Clinical response was observed at around 5 weeks in over 90% of patients with depression and approximately 75%-80% of those with anxiety, with remission achieved in about 60% and more than 50%, respectively. At 6 months, the response persisted in roughly 80% of participants, and remission was maintained in about two-thirds.
Mitchell et al. (2023) [9]	104 adults with moderate or severe post-traumatic stress disorder (PTSD)	MDMA (3 therapeutic sessions) + psychotherapy vs placebo + psychotherapy	CAPS-5 (primary endpoint), BDI-II, remission rates and measures of functional improvement	Mean change in CAPS-5 score was -23.7 points (95% CI: -26.94 to -20.44) in the MDMA group vs -14.8 points (95% CI: -18.28 to -11.28) in the placebo group (p < 0.001). A clinically significant improvement (≥10-point reduction) was achieved by 86.5% vs 69.0% of patients; 71.2% vs 47.6% no longer met PTSD criteria, and remission was observed in 46.2% vs 21.4%, respectively. Adverse events were predominantly mild or moderate.

Psilocybin in Substance Use Disorders

In the field of addiction treatment, the best documented data currently concern the use of psilocybin in the therapy of alcohol use disorder. Bogenschutz et al. conducted a randomised, double-blind trial with an active placebo in 95 adults with severe alcohol dependence, in which all participants received the same goal-oriented motivational therapy [22]. The pharmacological intervention consisted of two sessions with a high dose of psilocybin (25-40 mg/70 kg) or an active placebo (diphenhydramine; 50-100 mg). Over the 32-week follow-up, the percentage of heavy drinking days was 9.7% in the psilocybin group compared with 23.6% in the control group (difference: 13.9 percentage points; 95% CI: 3.0-24.7; p = 0.01), along with a significant reduction in the total number of alcohol units consumed per week relative to the placebo group. The safety profile was favourable, and no serious adverse reactions related to the study drug were recorded [22].

Data on nicotine dependence are methodologically weaker but suggest some therapeutic potential. Johnson et al. used two or three psilocybin sessions (20-30 mg/70 kg) combined with cognitive-behavioural therapy in 15 heavily dependent smokers [21]. At 12 months, abstinence was observed in 67% of participants, and in long-term follow-up (>16 months), it remained at 60%, as confirmed by objective measurements of carbon monoxide in exhaled air. The lack of a control group and the small sample size limit the possibility of drawing firm conclusions, although these findings provide an important rationale for designing randomized trials in the treatment of nicotine dependence [21].

Psilocybin for MDD

Davis et al. conducted a randomised trial with a wait-list control group in 27 adult patients with major depressive disorder (MDD), of whom 24 completed the full study protocol and follow-up visits with assessment of depressive symptom severity at weeks 1 and 4 [8]. The intervention consisted of two psilocybin sessions (20 mg at the first session and 30 mg at the second) combined with supportive psychotherapy. As early as one week after treatment, the mean score on the GRID-Hamilton Depression Rating Scale (GRID-HAMD) was approximately 8 points in the immediate-treatment group, compared with values of 23-24 points in the waiting-list group; 71% of patients met the criteria for clinical response (≥50% reduction in GRID-HAMD score), and 54% achieved remission (GRID-HAMD ≤7) at four weeks [8]. In a 12-month extended follow-up of the same patient cohort, Gukasyan et al. found that clinical response was maintained in 75% and remission in 58% of participants [27]. During this period, no serious adverse events judged to be related to psilocybin were reported and there were no reports of psilocybin use outside the study context, which points to a favourable long-term safety profile of this intervention in the analysed group [27].

Psilocybin vs. Escitalopram

Additional valuable data are provided by the six-month follow-up reported by Erritzoe et al., which extended a randomised, double-blind phase 2 trial comparing psilocybin with escitalopram, a selective serotonin reuptake inhibitor (SSRI) [26]. In the original study, 59 patients with MDD were assigned either to two sessions with a high dose of psilocybin (25 mg) or to six weeks of escitalopram treatment (10 mg/day for the first three weeks and 20 mg/day for the subsequent three weeks), with both groups receiving psychological support. The primary endpoint in the phase 2 trial was the change in depressive symptom severity measured by the Quick Inventory of Depressive Symptomatology-Self-Report (16-item version) (QIDS-SR-16) after six weeks, whereas the work by Erritzoe et al. examined the durability of effects after six months of follow-up [26]. The mean between-group difference in QIDS-SR-16 scores between psilocybin and escitalopram at six months was 1.51 points (95% CI: -1.35 to 4.38; p = 0.311), indicating no statistically significant differences in depressive symptom severity between the groups, with a clear maintenance of improvement relative to baseline in both conditions. However, analysis of secondary endpoints revealed some advantage of psilocybin over escitalopram: patients receiving psilocybin showed greater improvement in overall functioning (Work and Social Adjustment Scale (WSAS): mean between-group difference: -7.46; 95% CI: -12.4 to -2.47; p < 0.001), sense of meaning in life (Meaning in Life Questionnaire (MLQ): 4.86; 95% CI: 0.67-9.05; p = 0.021) and psychological connectedness (Watts’ Connectedness Scale (WCS): 11.02; 95% CI: 1.25-20.83; p = 0.033), which may suggest that this intervention has a broader impact than solely on depressive symptom severity [26].

Psilocybin in Cancer-Related Distress

Another important patient group comprises individuals with depression and anxiety in the context of cancer. Griffiths et al. randomised 51 people with advanced malignancy to receive, in two sessions, either a full dose of psilocybin (22-30 mg/70 kg) or a very low, placebo-like dose (1-3 mg/70 kg) in a cross-over design [23]. The main endpoints were clinical measures of depression (GRID-HAMD) and anxiety (Hamilton Anxiety Rating Scale (HAM-A)), assessed before treatment, five weeks after each session and at six months. The high dose produced a rapid and marked reduction in both depressive and anxiety symptoms. Approximately five weeks after the high-dose session, the proportion of patients meeting the criterion for clinical response (≥50% reduction in score versus baseline) exceeded 90% for depression and was around 75%-80% for anxiety in the group that received the high dose first, whereas values were significantly lower in the group starting with the low dose. At the same time, the remission rate (≥50% reduction in score and a total of ≤7 points on the relevant scale) after the high dose was about 60% for depression and more than 50% for anxiety. When both dose sequences were combined, the overall rate of clinical response at six months was around 80% for both depression and anxiety, with remission observed in approximately two-thirds of patients [23].

MDMA-Assisted Therapy for PTSD

With regard to PTSD, 3,4-methylenedioxymethamphetamine (MDMA) plays a key role. Mitchell et al. carried out a randomised, double-blind phase 3 trial in which 104 adults with moderate or severe PTSD were assigned to identical psychotherapeutic protocols combined with either MDMA or placebo [9]. Outcomes were assessed using the Clinician-Administered PTSD Scale for DSM-5 (CAPS-5), specifically the change from baseline to week 18 after the intervention. The mean reduction in CAPS-5 score was clearly greater in the MDMA group, 23.7 points (95% CI: -26.94 to -20.44) compared with 14.8 points (95% CI: -18.28 to -11.28) in the placebo group (p < 0.001). Change on the Sheehan Disability Scale (SDS), which evaluates functional impairment, also favoured the MDMA-assisted condition (mean change: -3.3 vs -2.1 points; p = 0.03). A clinically meaningful improvement, defined as at least a 10-point reduction in CAPS-5 score, was observed in 86.5% of participants receiving MDMA and 69.0% of those receiving placebo. By the end of the study, 71.2% versus 47.6% of patients, respectively, no longer met Diagnostic and Statistical Manual of Mental Disorders (Fifth Edition) (DSM-5) criteria for PTSD, and remission (loss of diagnosis and CAPS-5 ≤11) was found in 46.2% versus 21.4% of participants. Most adverse events were mild or moderate in intensity [9].

Safety considerations

The safety of psychedelic substances requires careful consideration; in particular, their potential interactions with concomitant medications and the range of acute and long-term psychiatric complications. Clinical trials involving classic psychedelics and MDMA suggest a relatively favourable safety profile in carefully selected patients treated under strict eligibility, monitoring and psychological support procedures [6,9]. A different picture emerges from case reports and review papers describing recreational, uncontrolled use of these substances [20,28].

Acute Adverse Events

In randomised clinical trials, short-term adverse events are usually mild to moderate and include transient increases in blood pressure and heart rate, nausea, headache, anxiety, dysphoria and episodes of "challenging" psychological experiences. In psilocybin trials, serious adverse events judged to be directly drug-related have been rare, and most disturbances resolved spontaneously within hours after the end of the session [8,23]. Similarly, in the study with MDMA, the most frequent adverse effects were transient autonomic symptoms (e.g. tachycardia, hypertension, increased jaw muscle tension), without events that were immediately life-threatening [9].

Drug-Drug Interactions

Potential drug-drug interactions between psychedelics and other medications, particularly serotonergic agents, are another key safety issue. A systematic review by Halman et al. indicates that co-administration with antidepressants, antipsychotics, anxiolytics or mood stabilisers may either attenuate or potentiate psychedelic effects [12]. The concomitant use of other serotonergic drugs (SSRIs, serotonin norepinephrine reuptake inhibitors (SNRIs), tricyclic antidepressants, MAO inhibitors) is of particular concern, as it may theoretically increase the risk of serotonin syndrome, even though reports of such events remain rare [12,20]. In the case of *ayahuasca*, which contains β-carboline MAO-A inhibitors, concomitant treatment with conventional antidepressants, sympathomimetics or other serotonergic substances is considered a major contraindication because of the heightened risk of hypertensive crises or serotonin toxicity [3,4]. For these reasons, clinical trial protocols usually include several-week medication washout periods and a detailed review of the patient’s current pharmacotherapy [6].

Flashbacks and HPPD

Recurrent episodes of sensations resembling the psychedelic experience after the acute effects have subsided (so-called flashbacks) represent a separate category of adverse effects. Studies in healthy volunteers suggest that short-lived, predominantly visual recurrences after LSD or psilocybin may occur in a few per cent of participants (up to around 9%) are usually mild, last from seconds to minutes and only rarely cause significant distress or functional impairment. Under controlled clinical conditions with carefully selected participants, this phenomenon is not regarded as a major clinical problem [12,29].

Hallucinogen-Persisting Perception Disorder (HPPD) is defined in DSM-5 as persistent or recurrent perceptual disturbances following prior use of hallucinogenic substances. Clinical descriptions and review papers indicate a predominance of visual symptoms: intensified after-images and trails, halos around objects, visual snow, distortions in size and shape and contrast disturbances such as unnaturally sharpened contours of surrounding objects [11,30]. HPPD occurs in two forms: an episodic form (type I), characterised by brief, sporadic recurrences of symptoms, and a chronic form (type II), in which perceptual disturbances are present continuously or almost continuously, often leading to marked distress and functional impairment [11]. In their systematic review of treatment options for HPPD, Neven and Blom highlight the very limited number of studies and the predominance of single case reports [30]. Benzodiazepines (especially clonazepam), antiepileptic drugs (e.g. lamotrigine) and, in some cases, atypical antipsychotics have been used most commonly, but their effectiveness is supported mainly by clinical experience rather than robust evidence. In addition, complete abstinence from psychoactive substances, stress reduction and psychotherapeutic support are routinely recommended [30].

Severe Psychiatric Complications

In a systematic review of published case reports, Yildirim et al. described the occurrence of schizophrenia-spectrum disorders, depressive and manic episodes and anxiety disorders, with some cases developing after a single exposure [28]. Although the authors emphasise that such complications are relatively rare, they often require prolonged inpatient treatment, and full recovery is not always achieved, particularly in psychotic disorders [28]. Schlag et al. note that most severe psychiatric complications have been associated with recreational use involving high doses, uncertain substance purity, concomitant use of other drugs and the absence of adequate support during the experience [20].

Contraindications and Patient Selection

Proposed contraindications to psychedelic-assisted therapy are largely derived from exclusion criteria used in clinical trials. The most commonly cited include current or past psychotic disorders, bipolar disorder type I, a family history of schizophrenia, severe personality disorders with a high risk of self-harm, active substance dependence (outside protocols specifically designed to treat that dependence), unstable cardiovascular disease, uncontrolled hypertension, serious cardiac arrhythmias, pregnancy and the use of medications with a high interaction potential, particularly MAO inhibitors [6,9,20]. Caution is also advised in patients with marked anxiety disorders and in younger individuals, in whom the long-term neuropsychological consequences of exposure to these agents are still insufficiently understood [20,28].

Risk Mitigation

An important determinant of both the effectiveness and the safety of psychedelic-assisted therapy is the "set and setting", that is, the combination of the patient’s internal psychological state (attitude, expectations, coping strategies) and the external environment in which the session takes place (safety of the setting, presence of trained staff) [2,19]. Clinical trials place strong emphasis on careful preparation, the development of a therapeutic alliance and post-session integration of the experience, which markedly reduces the incidence of acute anxiety reactions and the likelihood that any negative psychological effects will become persistent [8,9].

## Conclusions

Classical psychedelics and MDMA represent an important, although still insufficiently explored, component of potential modern therapies for psychiatric disorders. Their mechanism of action involves a direct effect on serotonin receptors, primarily 5-HT2A, accompanied by the induction of neuroplastic changes and the reorganization of functional brain networks, which may facilitate the modification of entrenched cognitive and emotional patterns. The subjective experiences associated with these substances, such as ego dissolution, mystical-type phenomena or a profound reinterpretation of internal content, appear to be an integral part of long-term clinical effects, particularly when combined with appropriately delivered psychotherapy and carefully planned “set and setting”.

The best-documented evidence concerns psilocybin in the treatment of depressive, anxiety disorders and alcohol use disorder. Most trials have demonstrated a rapid reduction in symptoms with benefits persisting for up to six to 12 months. It should be emphasized, however, that sample sizes have been small and patient populations highly selected. MDMA, used as an adjunct to psychotherapy, has shown clear superiority over psychotherapy alone in the treatment of moderate-to-severe PTSD, both in terms of symptom reduction and functional improvement. The United States Food and Drug Administration (FDA) has granted “breakthrough therapy” status to psilocybin- and MDMA-assisted therapies. This reflects the high expectations regarding their efficacy and the perceived need for accelerated clinical evaluation.

Review articles and clinical reports consistently stress the need for caution. Although reports of HPPD, psychotic disorders, affective complications and severe anxiety episodes are relatively rare, they demonstrate that risk is present, particularly in the context of recreational use without dose control, psychiatric supervision or professional support. Potential interactions with psychiatric medications, especially serotonergic agents and drugs affecting MAO activity, must also be taken into account.

In summary, the available data indicate a substantial, yet still only preliminarily documented, therapeutic potential of psychedelics and MDMA. Future research should focus on larger, multicentre trials with longer follow-up, direct comparisons with standard treatments and a more precise definition of patient groups most likely to achieve meaningful benefit at an acceptable level of risk. Only once these conditions are met, will it be possible to consider responsible integration of these interventions into routine clinical practice.
